# A Proteomic Platform Unveils the Brain Glycogen Phosphorylase as a Potential Therapeutic Target for Glioblastoma Multiforme

**DOI:** 10.3390/ijms23158200

**Published:** 2022-07-25

**Authors:** Giusy Ferraro, Matteo Mozzicafreddo, Roberta Ettari, Lorenzo Corsi, Maria Chiara Monti

**Affiliations:** 1 Department of Pharmacy, University of Salerno, 84084 Fisciano, Italy; gferraro@unisa.it; 2 PhD Program in Drug Discovery and Development, Department of Pharmacy, University of Salerno, 84084 Fisciano, Italy; 3 Department of Clinical and Molecular Sciences, Università Politecnica delle Marche, 60126 Ancona, Italy; m.mozzicafreddo@staff.univpm.it; 4 Department of Chemical, Biological, Pharmaceutical and Environmental Sciences, University of Messina, 98168 Messina, Italy; roberta.ettari@unime.it; 5 Department of Life Sciences, University of Modena and Reggio Emilia, 41125 Modena, Italy; 6 INBB (Istituto Nazionale di Biostrutture e Biosistemi), 00136 Roma, Italy

**Keywords:** glioblastoma multiforme, target identification, proteomics, 2,3-benzodiazepin-4-one, glycogen phosphorylase

## Abstract

In the last few years, several efforts have been made to identify original strategies against glioblastoma multiforme (GBM): this requires a more detailed investigation of the molecular mechanism of GBM so that novel targets can be identified for new possible therapeutic agents. Here, using a combined biochemical and proteomic approach, we evaluated the ability of a blood–brain barrier-permeable 2,3-benzodiazepin-4-one, called 1g, to interfere with the activity and the expression of brain glycogen phosphorylase (PYGB) on U87MG cell line in parallel with the capability of this compound to inhibit the cell growth and cycle. Thus, our results highlighted PYGB as a potential therapeutic target in GBM prompting 1g as a capable anticancer drug thanks to its ability to negatively modulate the uptake and metabolism of glucose, the so-called “Warburg effect”, whose increase is considered a common feature of cancer cells in respect of their normal counterparts.

## 1. Introduction

Glioblastoma multiforme (GBM) is the most common and aggressive malignant tumor of the Central Nervous System (SNC). Although the prognosis for patients affected by GBM is less unlucky by means of application combined of chemotherapy, radiotherapy and surgery, the median survival of patients is not increased significantly [1]. Recent studies began to explain the molecular pathway involved in GBM [2], such as RTK/Ras/PI3K, [3], TGF β signaling, Wnt [4], and endoplasmic reticulum (ER) stress ones [5], opening the road to new and more targeted therapeutic interventions characterized from a better effectiveness associated to a reduced toxicity.

It must also be emphasized that, although the novel biomarker-driven strategies mainly targeting the receptor tyrosine kinase [6] have been developed over the last decades, they have performed poorly during clinical trial testing. This is mainly due to low blood–brain barrier (BBB) permeability and increased resistance/tolerance of the small molecules under evaluation [7]. In this context, it must be underlined that the so-called “Warburg effect”, which is the ability of cancer cells to use glycolysis to provide energy, amino-acids and lipids, plays an important role in cancer growth and resistance [8]. Therefore, the modulation of glycogen metabolism is an important target for the pharmacological approach. 

**Figure 1:**
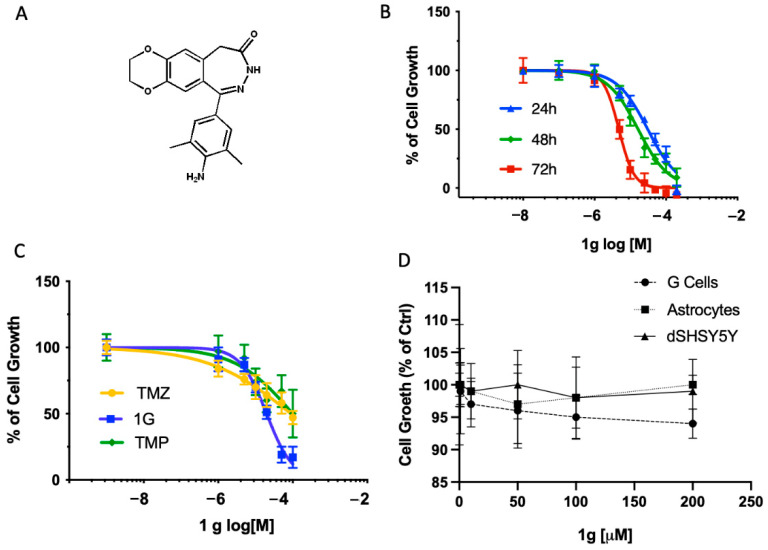
Cell viability of U87MG, dSHSY5Y, primary cerebellar granule neurons and primary astrocytes after pharmacological treatments. (**A**) 1g chemical structure; (**B**) Dose-response curves of 1g at different time points on U87MG; (**C**) Comparison of the inhibitory effect of 1g, TMZ and TMP on U87MG cell viability at 48 h; (**D**) Effects of 1g on primary cerebellar granule neurons and primary astrocytes after 48 h of incubation times at different concentrations. The values are expressed as % of control ± standard deviation (SD). GI_50_ is reported in the text.

Here, a very interesting and BBB-permeable compound endowed with multiple biological activities, the (1-(4-amino-3,5-dimethylphenyl)-3,5-dihydro-7,8-ethylenedioxy-4h-2,3-benzodiazepin-4-one) called 1g (Figure 1A), has been investigated [9]. This 2,3 benzodiazepin-4-one was able to inhibit leukemia Jurkat T cells growth, to arrest the transition of G2/M phase and to induce apoptosis [10]. In addition, 1g promoted a rapid and reversible reduction in microtubule growth, perturbing the formation of a functional mitotic spindle in human cells but not interfering directly with tubulin or perturbing microtubule assembly in vitro [11].

In our study, we evaluate the effect of 1g on different GBM cell lines and primary neuronal cells. In addition, a functional proteomics platform grounded on mass spectrometry-based Drug Affinity Response Target Stability (DARTS) has been spent to disclose the interactome of 1g on the glioblastoma U87MG cells which were chosen as protein sources. A functional proteomics platform based on DARTS and Limited Proteolysis (LiP) assisted by mass spectrometry has been implemented.

Nowadays, it is generally accepted that poly-pharmacological drugs profile can produce additive or synergistic actions while reducing side effects and significantly contribute to the high therapeutic success and, in this scenario, the functional proteomics-based identification of drug targets covers a key role [12]. Recently, new strategies have been developed and applied for the identification of the protein partners of free bioactive small molecules, such as SPROX (Stability of Proteins from Rates of Oxidation), TPP (Thermal Proteome Profiling), CETSA (Cellular Thermal Shift Assay) and DARTS, which has been applied in this paper [13,14]. The DARTS strategy functions due to the ability of a small molecule to increase the stability of the interacting target(s) towards enzymatic proteolysis; this particular behavior can be revealed by Sodium Dodecyl Sulfate-Polyacrylamide Gel Electrophoresis (SDS-PAGE) and the target(s) can be identified by a classical proteomics analysis. Moreover, a deeper analysis of the interaction profile between a small molecule and its specific targets can be performed through the recently developed strategy targeted-Limited Proteolysis-Mass Spectrometry (t-LiP-MS) approach. T-LiP-MS could be considered as a gel-free DARTS-like approach based on a double-protease digestion and a Multiple Reaction Monitoring (MRM)-MS detection, allowing the identification of protein conformational changes due to ligand/protein binding [15].

The 1g main target in the glioblastoma U87MG cells lysate is a receptor called Glycogen Phosphorylase, brain form (PYGB, also named GPBB). This is an enzyme involved in the regulation of carbohydrate metabolism by catalyzing the transformation from glycogen to glucose 1-phosphate [16,17]. Under different conditions, such as hypoglycemia and hypoxia, PYGB is released into cytosol from sarcoplasmic reticulum, it becomes active and it can supply glucose to the cell [17,18]. Interestingly, increased expression of PYGB has been reported in several cancers, such as ovarian cancer [19], hepatocellular carcinoma [20], colorectal cancer [21], prostate cancer [22] and non-small cell lung cancer [23]. Importantly, Zhao et al. [24] showed that PYG liver isoform (PYGL) was highly expressed in human gliomas, and it can be used as a predictive marker for poor prognosis [24]. All these findings suggest that PYG enzymes, and in particular PYGB, play an important role in cancer biology. However, so far, the involvement and or modulation of PYGB in GBM has not been studied.

## 2. Results

### 2.1. 1g Permeation by PAMPA Assays

Firstly, the behavior of 1g in the parallel artificial membrane permeability assay (PAMPA) has been tested to measure its effective permeability (expressed as −log Pe) through an artificial lipid membrane [25]. Propanolol and furosemide at 250 μM were used as positive and negative control molecules giving a −log Pe of 5.30 and 6.64, respectively (−log Pe < 6 is considered good permeability and −log Pe ˃ 6.5 as impermeable). In this assay, 1g displayed a good propensity to cross the membrane in vitro with a −log Pe of 5.22 ± 0.04.

### 2.2. Effects of 1g on Cell Growth in U87MG, Neuronal Differentiated SHSY5Y and Primary Neuronal Culture

To determine a possible specific activity of 1g derivative on glioblastoma cell line, we evaluated its growth inhibition effect on different cell cultures, such as primary neuronal cerebellar granule cells, astrocyte primary cells and the differentiated neuroblastoma cell line SHSY5Y (dSHSY5Y). 1g was able to significantly inhibit the cell growth in a dose and time-dependent manner in U87MG cell line (Figure 1B) in comparison to the cells treated with the vehicle alone (0.1% DMSO). Indeed, the GI_50_ ranged from 30 ± 3 µM, 17 ± 3 µM to 4 ± 2 µM at 24, 48 and 72 h, respectively. Noteworthy, 1g produced a greater inhibition at 48 h of treatment when compared to the temozolomide (TMZ) and Talampanel (TMP) used as a reference drug. In particular, the GI_50_ of 1g was about five times less than that measured with TMZ (89 ± 5 µM) and TMP (99 ± 12 µM), as indicated in Figure 1C.

Differently, as shown in Figure 1D, 1g was unable to modulate the cell proliferation of granule neurons, astrocytes and dSHSY5Y at all the concentrations tested after 48 h of incubation (Figure 1D), suggesting that the compound did not affect well differentiated neurons and low proliferating cells.

### 2.3. Cell Cycle Analysis 

**Figure 2:**
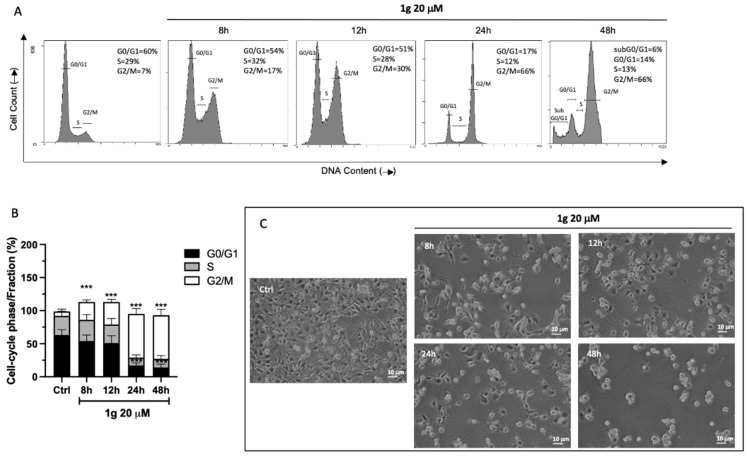
Effect of 1g on U87MG cell cycle. (**A**) Representative cell cycle distributions assessed by propidium iodide staining and 5′-bromo-2′-deoxyuridine BrdU incorporation in enzymatically disaggregate U87MG cells treated with 20 µM of 1g at the indicated time points. (**B**) The percentages of cells in G0/G1, S and G2/M phases are presented in the histograms. Data are expressed as the mean ± SD obtained from three independent experiments performed in duplicate. The values are expressed as the mean ± SD of three independent experiments (*n* = 4 per group). *** *p* < 0.001 vs. untreated cells (Ctrl), using a one-way ANOVA with Dunnett’s as post-test. (**C**) Light microscopic micrographs at 20× magnification of U87MG treated or not with 1g 20 µM at different time points.

We then evaluated the cell cycle distribution of U87MG cells at different time points (8, 12, 24 h) using a concentration of 1g corresponding to the GI_50_ obtained after 24 h of incubation time. Flow cytometry analysis showed that 1g was able to induce a cells accumulation in G2/M phase in a time-dependent fashion (Figure 2A), reaching a maximum response after 24 and 48 h of incubation time. In particular, 1g produced a significant increase of percentage of G2/M arrest already after 8 h of incubation, from 6.67 ± 3.67% in the control cells to 27 ± 3.3%, increasing over time and reaching 34.1 ± 4.0% at 12 h and 66.3 ± 5.0% of G2/M arrest at both 24 and 48 h. (Figure 2B). In parallel, we assisted to a progressive decrease in the percentage of cells in the G0/G1 phase from 63.1 ± 4.2 % to 54.1 ± 3.1% at 8 h of treatment and to 16.6 ± 5% and 14 ± 5% after 24 h and 48 h of incubation time, respectively (Figure 2B). Interestingly, the cell line change morphology after the first 8 h of 1g treatment, becoming rounded and they maintain that structural conformation over time (Figure 2C).

### 2.4. Protein Expression Evaluation

**Figure 3:**
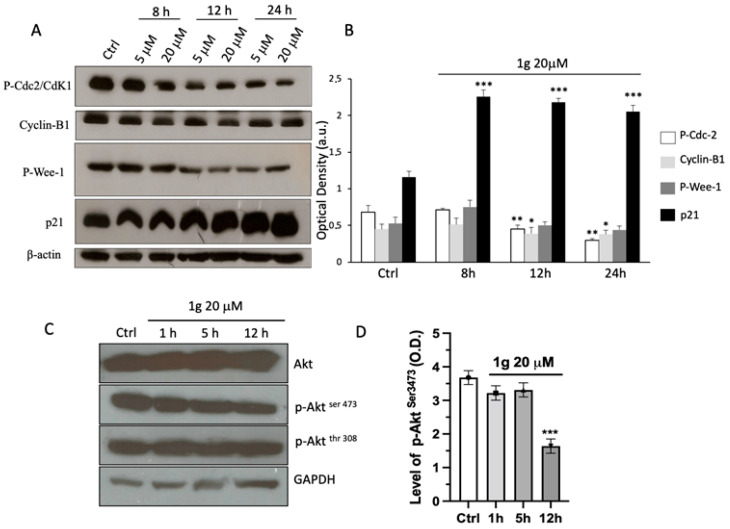
Effect of 1g on P-Cdc2, cyclin-B1, P-Wee-1 p21waf/cip1, Akt, p-Akt (Ser473) and p-Akt (Thr308) protein expression in U87MG cells. (**A**) Representative Western blots of P-Cdc2, cyclin-B1, P-Wee-1 and p21waf/cip1 at different times and concentrations and (**B**) densitometric analyses of protein levels of P-Cdc2, cyclin-B1, P-Wee-1 and p21waf/cip1. (**C**) Representative Western blots at different time points of Akt, p-Akt (Ser473) and p-Akt (Thr308) of U87MG cell lysate after incubation with 20 μM of 1g for 1, 5 and 12 h. (**D**) Densitometric analyses of protein levels of Akt, p-Akt (Ser473) of U87MG cell lysate after incubation with 20 μM of 1g for 1–5 and 12 h. Densitometry values were normalized to the protein loading control, beta-actin or Glyceraldehyde 3-phosphate dehydrogenase (GAPDH). The values are expressed as the mean ± SD of three independent experiments (*n* = 4 per group). * *p* < 0.01, ** *p* < 0.005, *** *p* < 0.001 vs. untreated cells (Ctrl), using a one-way ANOVA with Dunnett’s as post-test.

In order to get more inside in the 1g induced G2/M arrest, several cell-cycle determinants directly involved in the G2/M phase transition, such as cyclin B1, phospho-Cdc2 (Tyr15), phospho-Wee-1 (Ser642), and p21Waf/Cip1, were evaluated by immunoblot analysis. Figure 3A shows a decrease of protein levels of phospho-Wee-1 and phospho-Cdc-2 after 12 h, reaching significant values after 24 h in cell treated with 1g at 20 μM compared to the control (Figure 3B). Cyclin B1 immunoreactivity, instead, did not change significantly at all the time points tested. Interestingly, the level of the endogenous p21 Waf/Cip1 protein increased significantly already after 8 h of treatment, reaching a maximal induction between 12 and 24 h, confirming the block of the mitotic activity. Similarly, the data of Akt protein expression showed that the 1g was able to modulate the phosphorylation of this protein. Indeed, the incubation with 20 μM of 1g decreased significantly the intensity of the p-Akt (Ser473) after 12 h of incubation time, while the expression of non-phosphorylated Akt did not show any variation (Figure 3C,D). After 1 and 5 h of incubation, no change in immunoreactivity for all tested protein has been detected. Interestingly, 1g was unable to modulate the expression of p-Akt (Thr308) (Figure 3C,D), indicating a specific interaction toward the phosphorylation at Ser473.

### 2.5. Target Identification of 1g by a Proteomic Platform

In order to investigate the molecular mechanism of action of 1g on U87MG cells, we move to apply our novel proteomic platform on this interesting system: in particular, our combined multidisciplinary approach can be summarized in the following steps: (a) identification of 1g interactome by DARTS coupled to high resolution mass spectrometry, bioinformatics and Western blotting analysis to validate the results; (b) t-LiP MRM analysis to look over at the interaction features between 1g and its main target and (c) molecular docking of the complex between 1g and its most reliable protein partner.

#### 2.5.1. Identification of 1g Cellular Partner(s) through DARTS

A typical DARTS experiment initiates with the controlled proteolysis of a cellular lysate, pre-treated or not with the small molecule, with the low-specificity protease subtilisin under native conditions. The following SDS-PAGE of the samples allows to monitor proteins’ resistance to the enzymatic hydrolysis: the lane intensity corresponding to the putative protein target(s) will raise in the samples pre-treated with the small molecule, due to its protective effect, in a concentration dependent fashion. Thus, the target protein(s) can be identified through classical proteomic approaches by in situ digestion, nano-Ultra Performance Liquid Chromatography (UPLC)-MS/MS experiments and the use of bioinformatics tools. 

**Figure 4:**
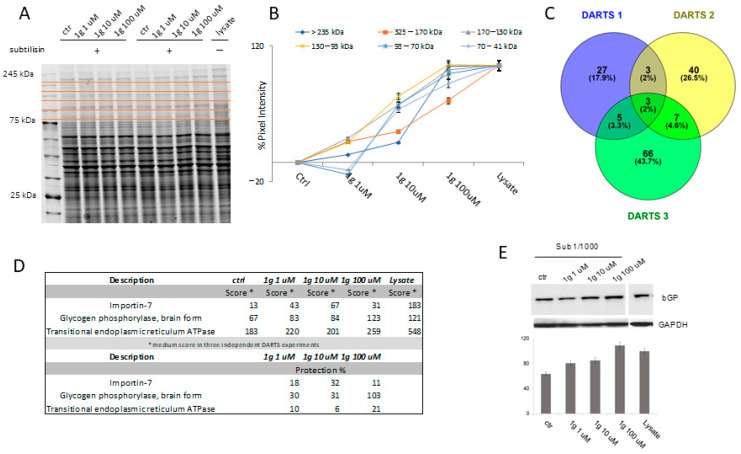
(**A**) Coomassie stained SDS-PAGE of a DARTS experiment performed with increasing 1g amounts. Red lines indicate the gel cutting pattern. (**B**) Densitometric analysis of the SDS-PAGE as the pixel intensity of each gel region *versus* molecular weight. The major variation of pixel intensity of 1g-treated samples can be observed at molecular weights (MW) higher between 130 and 93 kDa. (**C**) Venn diagram of 1g protected proteins identified in three independent DARTS experiments. Three of them were protected in all performed assays. (**D**) Filtered list of 1g putative partners, reported with their protection (%) from proteolysis in DARTS biological replicates. (**E**) Western blotting and densitometric analysis of one DARTS experiment showing increasing 1g amounts shelter PYGB from proteolysis (95 kDa signals). GAPDH has been used as a loading normalizer.

In our experiments, U87MG cell samples lysed in mild non-denaturing conditions were incubated with increasing 1g concentrations, except one treated with the vehicle and representing the negative control, and then subjected to subtilisin-mediated limited proteolysis. An undigested lysate sample without 1g was kept as a positive control. Then, all samples were submitted to SDS-PAGE separation and revealed by Coomassie blue and the gel lanes were carefully excised and digested, principally those bands whose intensity raised up at increasing 1g concentrations (Figure 4A,B). The nano-UPLC-MS/MS analysis of the digested peptide samples, followed by a Mascot database search, gave proteins identification. All the experiments were carried out in triplicate: proteins identified in all DARTS were considered (Figure 4C,D and Figure S1) to identify 1g interacting ones by comparing the Mascot Score outputs with both the positive and negative control samples. Thus, 1g protection levels (reported as percentages) were evaluated for each identified protein: among them, PYGB has been selected as the main and most reliable 1g partner, since it was better protected from proteolysis in all DARTS replicates (Figure 4D). The direct interaction between the protein and the small molecule was then unequivocally determined by Western blotting analysis, submitting all DARTS samples to an anti-PYGB antibody reaction (Figure 4E). In fact, from the comparison of the immunoblotting signals corresponding to undigested PYGB (97 kDa bands), it emerges that the intact protein signal increases its intensity accordingly to 1g concentration. An accurate densitometric analysis was carried out on the full-length PYGB signal, using GAPDH as loading normalizer (Figure 4E).

#### 2.5.2. Analysis of the Interaction Features at the Basis of 1g-PYGB Binding by t-LiP MRM

**Figure 5:**
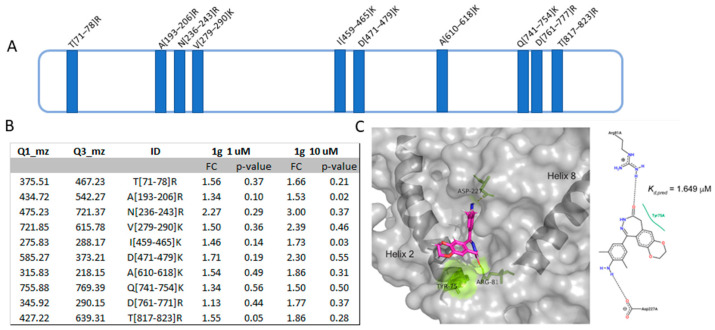
(**A**) Schematic PYGB cartoon is depicted in azure, with the 1g-protected peptides highlighted in blue. (**B**) Selected PYGB peptides reported with their parent and daughter *m/z* values, their length and the calculated fold changes (FC) due to 1g protection. *p*-values have been calculated and only tryptic peptides with a *p* ≤ 0.5 are reported. (**C**) Best predicted docking pose of 1g (in violet sticks) on PYGB. The amino acids involved in the interaction are reported in the 2D representation and as yellow sticks in the 3D model. The AMP binding site containing 1g is delimited by the two helices in the cartoon representation.

To further explore the interaction profile of 1g, our optimized t-LiP MRM strategy has been carried out. t-LiP MRM lets the recognition of the target/ligand interface region(s) in a crude cell lysate, looking at the protein conformational alterations due to a ligand binding. The native proteins from U87MG cells were treated or not with 1g and a double-protease digestion was performed: first, a subtilisin limited proteolysis step was achieved under native controlled conditions and, then, a full tryptic digestion was finalized in a denaturing setting. This sequential treatment generates a combination of semi-tryptic and fully tryptic peptides, the latter suitable for targeted MRM-MS quantification analysis. Indeed, the area of fully tryptic peptides peaks can be considered indicative of the local target structural changes due to ligand binding: it will be higher when subtilisin limited proteolysis is less effective due to small molecule covering attackable protein regions. Initially, an in-silico search using the bio-informatics device PeptideAtlas and SRMAtlas set the more likely MRM transitions of PYGB theoretical fully tryptic peptides to map the protein, then a cell lysate sample was denatured and extensively proteolyzed by trypsin to unequivocally recognize the most reliable peptide signals and their most intense daughter ions by LC-MRM-MS (Figure S2). Next, native protein mixtures were incubated with 1g (1 and 10 μM) or vehicle (negative control) and treated with subtilisin in restricted conditions of time, temperature and at an enzyme-to-protein ratio of 1:1500 (*w/w*). Subtilisin was then quenched and all of the samples were denatured and fully digested by trypsin and the mixtures were run on the LC-MRM-MS system to quantify the area of each PYGB tryptic peptide. Then, peptides mapping for PYGB regions sensitive to subtilisin (also called LiP peptides) were selected as the ones whose area was significantly inferior in the doubly digested sample (negative control) compared to the trypsin-only digested sample (positive control). Next, these peptides were examined in the controls and 1g treated samples: LiP peptides with an increased intensity in the samples exposed to the small molecule were considered symptomatic of 1g protection on specific PYGB region(s) (Figure 5A,B, Figure S3). In particular, PYGB conformational changes were identified on the peptide 71–78, in the region from 193 to 290, on the peptide 610–618 and from 459 to 479 and from 761 to 823, as shown in Figure 5A,B. The fold changes reported in Figure 5B represent the grade of protection due to 1g interaction: they have been calculated as the ratio between the area of the tryptic peptide in the 1g-treated sample and in the untreated sample. The same t-LIP experiments have been repeated three times and the fold changes were calculated over the means of the peptides area. As reported by the group of Prof. Fernando Rodrigues-Lima [26], PYGB monomer is composed of two domains: the N-terminal domain from residues 22 to 484 and the C-terminal domain from residues 485 to 822 bearing the catalytic site at their interface. PYGB is found as an equilibrium between a monomeric and dimeric form, which is shift toward the dimeric state upon activation. The association of two PYGB monomers into the functional dimer involves helix 2 (residues 49–77), the β4/β5 loop (residues 180–198), the cap loop (residues 41–48) and the tower helices (residues 259–278). The helices are connected to the gate loop (residues 279–289). The gate loop adopts either an open or a closed configuration depending the activation state of the enzyme (active or inactive). Moreover, the side chain of Tyr196 from the β4/β5 loop is involved in the binding with the phosphate of AMP as well as the nucleotide moiety of AMP interacts weakly with helix 2, forming co-planar stacking with the side chain of Tyr75. The conformation of Tyr75 in PYGB is stabilized by the formation of a hydrogen bond between the phosphate group of AMP and Tyr196, which contributes to the geometry of AMP in PYGB by favoring the co-planar stacking between Tyr75 and the nucleotide part of AMP. 

Thus, looking at t-LIP-MRM data, 1g seems to affect the interface between PYGB monomers and the so-called ‘gate loop’, since both peptides 71–78 and 279–290 are protected, thus altering the dimerization process. Moreover, since Tyr75 and Tyr196 are in t-LIP protected peptides, namely at amino acids 71–78 and 193–206, it is plausible that 1 g also affects the binding of AMP, a known PYGB positive modulator. 

#### 2.5.3. Molecular Docking Analysis of PYGB/1g Complex

In order to provide information on the molecular basis of PYGB/1g interaction, a docking simulation has been carried out. Figure 5C reports the best docking geometry of 1g into the AMP binding site of PYGB which is at the basis of the minimum predicted equilibrium dissociation constant (Kdpred) of 1.649 µM, obtained from ΔG = −7.884 kcal mol^−1^; this value is at least one order of magnitude lower than the dissociation constant for the first AMP ligand bound to enzyme dimer [27]. In detail, this geometry contemplates the formation of two H-bonds of 1g with Arg81 and Asp227 and a hydrophobic contact with Tyr75, fully fitting with t-LIP-MS data.

### 2.6. 1g Negatively Affected PYGB Expression and Activity

**Figure 6:**
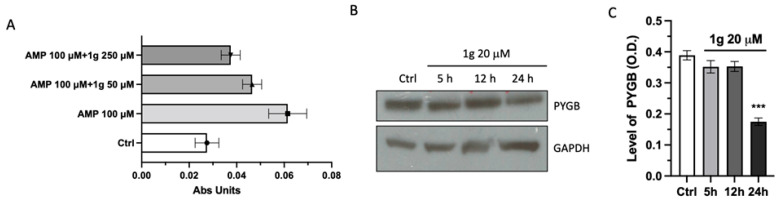
Effect of 1g and AMP on the PYGB activity and expression in U87MG cell lysates. (**A**) The Abs Units Increment is the variation in the absorbance of the sample after 30 min of reaction. S.D. was calculated on three independent measurements. (**B**) Representative Western blots of PYGB at different time points. (**C**) Densitometric analyses of protein levels of PYGB, of U87MG cell lysate after incubation with 20 µM of 1g for 5–12 and 24 h. *** *p* < 0.001 vs. untreated cells (Ctrl), using a one-way ANOVA with Dunnett’s as post-test.

Then, an in vitro activity assay has been equipped aiming to explore the effect of 1 g on PYGB by measuring the glycogenolytic activity of this enzyme in presence or not of different concentrations of 1g and/or AMP, the endogenous PYGB positive effector. To simplify the detection of variations in the PYGB-dependent glycogenolysis, this reaction has been coupled with the phosphoglucomutase-mediated conversion of its product, the glucose-1-phosphate into glucose-6-phosphate, which is converted by glucose-6-phosphate dehydrogenase into the corresponding lactone. The last reaction of this enzymatic chain is a Nicotinamide Adenine Dinucleotide Phosphate (NADP+)-dependent transformation also generating NADPH as product. Thus, the PYGB activity has been monitored by measuring the Optical Density (OD) at 340 nm and corresponding to the amount of the produced NADPH. The spectrophotometric assay has been performed in a kinetic mode and the histogram in Figure 6A has been obtained by calculating the variation of Absorbance (Abs) with respect to an opportune blank for each experimental condition. The trend of this activity assay means that 1g modulates in a negative mode the activity of PYGB. 

Finally, the analysis of protein expression of PYGB on U87MG cell line revealed that 1 g treatment was able to reduce the band intensity in a time-dependent manner. Indeed, as shown in Figure 6B,C, the protein level of cells treated with 20 µM of 1 g decreases significantly after 24 h of incubation time, whereas after 5 and 12 h, the reduction of immunoreactivity was not present.

## 3. Discussion

In the last few years, several new approaches have been proposed for the treatment of GBM, such as immunotherapy and the use of anti-cancer drugs targeting over-expressed Receptor tyrosine kinases (RTKs) proteins [28]. Regrettably, only few patients respond to these therapies due to the cell resistance and the ability of GBM cells to escape the detection by immune cells [29]. 

The research of new strategies entails a better knowledge of the molecular mechanism of GBM so that new possible targets can be identified for novel therapeutic agents. In this contest, a common feature of cancer cells distinguishing them from their normal counterparts is the increased uptake and metabolism of glucose, the so-called “Warburg effect” (Warburg 1956). Several studies have been completed on both the molecular mechanisms underlying the Warburg effect and its cellular function, including those on glycogen metabolism [30]. Particularly, in the last decade, scientific attention has been paid to the glycogen turnover in tumor cells [31] and on the glycogen PYGs role.

Here, using a novel proteomic approach, we evaluated the ability of 1g to interfere with the activity and the protein expression of PYGB on U87MG cell line in parallel with the capability of the compound to inhibit the cell growth. The data obtained on cell viability inhibition clearly showed that the efficacy 1g is greater than TMP and TMZ. Although this result, per se, did not predict clinical efficacy, the increased pharmacological potency displayed by 1g in comparison to TMZ is really noteworthy. This result acquires even greater significance considering the no-effect of 1g on differentiated dSHSY5Y, suggesting a possible specific action on hyperproliferating cells. In particular, 1g was able to arrest the GBM cell cycle in the G2/M phase, confirming our previous data [10]. Therefore, we evaluated the protein expression of the major complex involved in the G2/M transition checkpoint such as CDK1/Cyclin B1. The analysis of cyclin B1, of the inactive phosphorylated form of Cdc2(Tyr15)/CDK1 and the inactive phosphorylated form of the regulatory protein Wee-1 showed that 1g was able to decrease the expression of CyclinB1 and p-Cdc2(Tyr15), at 12 and 24 h of treatment, whereas it was capable only to slightly decrease the p-Wee-1(Ser642) protein expression over time. Moreover, analysis of p21 revealed that the protein level was dramatically increase already after 8 h of treatment and lasting after 12 and 24 h, indicating an important role of the protein in the 1g-mediated cell cycle arrest. Since our previous results, although performed on different cells, did not detect significant amount p21 in the 1g treated cells, it is reasonable that the increase of this protein is an epiphenomenon rather than a direct target of the molecule. We then performed a proteomic analysis to find out a range of possible protein targets interacting with 1g. In detail, DARTS experiments disclosed three more affine targets which were stabilized by 1g from the proteolytic action of a non-specific enzyme. In between these, PYGB is the best protected at all 1 g tested concentrations, as also detected by immunoblotting. Moreover, thanks to limited proteolysis and molecular docking analysis, it has been revealed that 1g interacts well inside the AMP recognition hole, establishing favorable contacts with the amino acids into the AMP binding site. 

As mentioned above, glycogen metabolism plays an important role in the cancer progression [32]. Several lines of evidence suggest that the different isoforms of PYGs are over-expressed and involved in the maintenance [33], progression and invasiveness [34,35] of different cancer cells. In particular, PYGB has been reported to implicated both in tumor progression of gastric cancer through the modulation of Wnt/β-catenin pathway [35] and in the invasion of breast cancer cells [34]. In addition, Zhan at al. showed that PYGB activates the PI3k/Akt signaling pathway and, therefore, the tumorigenesis of non-small cell lung cancer [36].

The inhibition of PYGB activity and protein expression by 1g measured in the biochemical assays reported in this paper is well related to a reduction of U87MG cell proliferation both due to a decrease of cancer cell fuel and the modulation of the PI3k/Akt pathway [37]. Indeed, the inhibition of PI3k/Akt signal with the specific inhibitors LY 294002 or genistein also arrests the cell cycle in G2/M phase [37] by increasing p21 expression [38]. Our results seem to confirm this finding; indeed, 1g decreased the expression of p-Akt (Ser473), not altering the non-phosphorylated Akt and the p-Akt (Thr308). This result is interesting since the phosphorylation site at Ser473 has been identified as mammalian target of rapamycin (mTOR) [39,40] ant it is over activated in cancer cells.

In this context, our results support the hypothesis that the phosphorylation at Ser473 could be an upstream site-specific regulation of Akt pathway. The downregulation of p-Akt (Ser473) might be attributable to the modulation of PYGB activity elicited by 1g rather than a consequence of the inhibition of PYGB protein expression, which occurs only after long time.

## 4. Materials and Methods

### 4.1. 1g PAMPA Assays

Donor solution (250 μM) was prepared by diluting 5 mM 1g stock solution in Dimethyl sulfoxide (DMSO) using phosphate buffer (pH 7.4, 0.01 M). Filter membranes were coated with 5 μL of specific lipid solution prepared a 1% phosphatidylcholine solution in n-dodecane. Donor solution (150 μL) was added to each well of the filter plate. To each well of the acceptor plate, 300 μL of solution (5% DMSO in phosphate buffer) was added. 1 g was tested in triplicate. The sandwich was incubated for 24 h at room temperature under gentle shaking. After the incubation time, the sandwich plates were separated and 250 μL of the acceptor plate and 100 μL of donor were transferred to a UV quartz microtiter plate and measured by UV spectroscopy, using a Multiscan GO microplate spectrophotometer (Thermo Scientific, Waltham, MA, USA) at 250−500 nm at step of 5 nm. Reference solutions (250 μL) were prepared diluting the sample stock solutions to the same concentration as that with no membrane barrier. The permeability value Log Pe has been determined as reported in [25]. The integrity of the membrane was checked using propanolol and furosemide as control molecules.

### 4.2. Cell Cultures 

Primary cultures of rat cerebellar granule neurons and astrocytes were prepared from 8-day-old Sprague-Dawley rat cerebella as previously described [41]. Briefly, both primary granule cells and astrocytes were dispersed with trypsin (0.25 mg/mL; Sigma, Milan, Italy) and plated at a density of 100–105 cells/cm^2^ on 96 multi wells plate coated with poly-L-lysine (10 mg/mL; Merck Life Science, Milan, Italy). Cells were cultured in DMEM (Euroclone, Pero, Milan, Italy) supplemented with 10% FBS, 2 mM glutamine, and 100 mg/mL of gentamycin (Euroclone, Pero, Milan, Italy). In the cerebellar granule cells, 1 mM of cytosine arabinofuranoside (Ara-C; Merck Life Science, Milan, Italy) was added 18–24 h after plated to inhibit glial proliferation. Culture medium was replaced at days in vitro (DIV) 7 and 10, and the confluent cultures used at DIV 11.

Human cell line U87MG (ATCC), were cultured at 10–105 cells/cm^2^ on 96 multiwell plates in EMEM supplemented with 4 mM L-glutamine, 100 U/mL penicillin, 100 mg/mL streptomycin, 1% sodium pyruvate, 1% non-essential amino acids, and 10% FBS. Human neuroblastoma SH-SY5Y cells (ATCC) were grown at 10–105 cells/cm^2^ on 96 multiwell plates, in a mixture of 1:1 of Ham’s F12 and DMEM supplemented with 10% FBS, 2 mM L-glutamine, 100 U/mL penicillin and 100 μg/mL streptomycin. The cells were then differentiated into neuron-like population by adding retinoic acid (10 µM) for 10 days and used at DIV 12. The cells were all maintained at 37 °C in 5% CO_2_. Cells were visualized with Nikon Inverted Microscope Eclipse Ti-E equipped with a Digital Sight camera DS-Qi2 (Nikon Instruments, Tokyo, Japan) and images were acquired with NIS-Elements (Nikon Instruments, Tokyo, Japan) software.

### 4.3. Determination of Cell Growth Inhibition

1 g, TMP and TMZ (Sigma) were initially dissolved in DMSO at concentration of 100 mM, and serial dilutions were then prepared in culture medium, so that the final concentration of DMSO was <0.1%. Cell proliferation was assessed after 24–48–72 h of continuous exposure with different concentrations of the compounds (0.1–200 µM) using CCK-8 assay (Dojindo Laboratories, Kumamoto, Japan). Briefly, the different cell lines were plated on 96-well plates (Euroclone) at concentrations described above. After exposure to desired concentrations of the different compounds, 10 µL CCK-8 was added to each well and incubated for of 2.5 h at 37 °C. The absorption was measured at 450 nm using a multiplate reader multiscan FC (Thermo Scientific). The percentage growth was calculated using the following calculation: % growth = 100 × [(T − T0)/(C − T0)] where (T) was the growth of the cells in presence of the compound at different concentrations and at a specific time point, (T0) represents the number of cells at the time 0 of the experiment and (C) the growth of the control at a specific time point. The inhibition that reduces the cell population by 50% (GI_50_) was calculated using GraphPad Prism 6 (Graph-Pad 9 Software Inc., San Diego, CA, USA). 

### 4.4. Cell Cycle Analysis

U87MG were seeded at a density of approximately 90,000 cells/cm^2^ into 6-well plates, cultured overnight and treated with 20 µM of 1g or 0.1% DMSO (control). Following 8–12–24 h of incubation, cells were harvested, washed with saline phosphate buffer (PBS) and the different phases of cell cycle was analyzed by BrdU/PI staining. Evaluation of the different phases of cell cycle was analyzed by BrdU/PI staining performed as described by Manfredini et al. [42]. Briefly, cells were pre-incubated with 10 µM BrdU (Sigma Aldrich) and stained with a purified mouse primary monoclonal antibody directed against BrdU (BD Biosciences, Allschwil, UK) followed by a rabbit anti-mouse immunoglobulin IgG secondary antibody conjugated with fluorescein isothiocyanate (FITC) (Dako A⁄S, Hilden, Germany). Samples were then resuspended in a 50 µg/mL PI water solution. Both the assays were analyzed by Coulter Epics XL flow cytometer (Coulter Electronics Inc., Miami, FL, USA).

### 4.5. Western Blot Analysis 

Immunoblot was performed as already described by [43]. Briefly, proteins from U87MG treated or not with different concentrations of 1g at different time points were extracted by lysing and homogenized (in ice) the samples in RIPA buffer (50 mM Tris-HCl pH 7.4, 150 mM NaCl, 1% Na deoxycolate, 1% Triton X-100, 2 mM PMSF, Merck Life Science S.r.l). The protein concentrations were quantified using Bradford colorimetric method (Pierce, Rockford, IL, USA) according to the manufacturer’s instruction. An equal amount of protein, 0.5 µg/µL for each sample, was loaded onto a pre-cast 12% SDS-PAGE (Thermofisher Invitrogen, Waltham, MA, USA) and transferred to nitrocellulose membrane (Thermofisher Invitrogen). Membrane was blocked in TBST buffer (20 mM Tris-HCl, 0.5 M NaCl and 0.05% Tween 20) containing 5% non-fat dried or 5% BSA and incubated with primary anti-cyclin B1 (1:1000), anti-cdc-2 (1:1000), anti-phospho-cdc2(Tyr15) (1:1000), anti-p21 Waf/Cip1 (1:1000), anti-pospho-Wee-1(Ser642) (1:1000), anti-PYGB (1:1000), Anti-Akt (1:1000), Anti-phospho-Akt(Ser473) (1:2000) and Anti-phospho-Akt(Thr308) (1:1000) (I Thermofisher Invitrogen), at 5 °C overnight under agitation. Membrane was then washed 3 times in TBST, incubated for 1.5 h with HRP-conjugated anti-mouse or anti-rabbit antibody (Cell Signaling, Danvers, MA, USA) and visualized using chemiluminescence method (GE Healthcare, Amersham, UK). The determination of relative immunocomplexes was performed using densitometric analysis using a BioRad GS 690 Imaging densitometer with molecular analysis software (Version 8.1, Life science, Milan, Italy). Β-actin or GAPDH was used a loading control. For the uncropped images see Figures S4, S5 and S7.

### 4.6. Identification of 1g Cellular Targets

U87MG cells were lysed in PBS 0.1% Igepal in presence of a protease inhibitor mixture (Sigma Aldrich). Lysate was centrifuged at 10,000× *g* for 10 min and the protein concentration of the supernatant was determined by Bradford assay. DARTS experiments were performed as reported: different amounts of unmodified 1g (1 μM, 10 μM and 100 μM) were incubated with 300 μg of U87MG cell lysate for 1 h at room temperature. The obtained samples were then submitted to limited proteolysis, for 30 min at 25 °C, with subtilisin (Sigma Aldrich) at different concentrations. The best subtilisin defect has been evaluated as 1/1000 *w/w* in respect of proteins amount. Two samples of cell lysate were solely treated with DMSO and one of them with subtilisin, as control experiments. Then, the protease was quenched by adding phenylmethylsulfonyl fluoride (PMSF, Sigma-Aldrich, St. Louis, MO, USA, 1 mM final concentration) to each sample. Subsequently, all samples were boiled in SDS-PAGE loading buffer and 20 μg were loaded on a 4–12% Bis-Tris Criterion TM XT Precast Gel (Bio-Rad Laboratories S.r.l., Hercules, CA, USA), which was then stained with a Comassie solution and submitted to a densitometric analysis through ImageJ. This experiment was carried out in triplicate. Protein bands were excised from the gels and submitted to an in situ tryptic digestion protocol [44]. Briefly, gel slices were reduced with DTT (1,4-dithiothreitol), alkylated with IAA (iodoacetamide), washed, and rehydrated on ice for 1 h in a 12 ng/µL trypsin solution. Then, the enzyme excess was removed and replaced with ammonium bicarbonate (AmBic, 50 mM, pH 8.5), allowing protein digestion to proceed overnight at 37 °C. Subsequently, supernatants were collected and peptides were extracted from each gel slice, shrinking them in 100% ACN (acetonitrile). The obtained peptides mixtures were dried and dissolved in 10% FA for the subsequent nano-UPLC-MS/MS analysis on a Q-Exactive Classic Mass Spectrometer coupled to an UltiMate 3000 Ultra-High-Pressure Liquid Chromatography (nano-UPLC) system, equipped with an EASY-Spray PepMAP^TM^ RSLC C18 column (3 µm, 100 Å, 75 µm × 50 cm, ThermoFisher Scientific). Peptides elution was achieved at a flow rate of 300 nL/min with the following gradient: 1 min at 3% B, 1–50 min at 45% B, 50 min–51 min at 80% B, 51–54 min at 80% B, 55 min back at 3% B, until 61 min (A: 0.1% AcOH, 95% H_2_O, 5% ACN; B: 0.1% AcOH, 95% ACN, 5% H_2_O). The mass spectrometer was operated in data-dependent acquisition mode. Full scan MS spectra were acquired with the following settings: scan range 375–1500 *m/z*, full-scan automatic gain control (AGC) target 3 × 10^6^ at 70,000 resolution, and maximum injection time 50 ms. MS2 spectra were generated for up to 10 precursors (normalized collision energy of 28%); fragment ions were acquired at a 17,500 resolution with an AGC target of 1 × 10^5^ and a maximum injection time of 50 ms. Subsequently, database searches were carried out on Mascot Deamon, employing the SwissProt database and the following settings: two missed cleavages; carbamidomethyl (C) as fixed modification; oxidation (M) and phosphorylation (ST) as variable modifications; peptide tolerance 30 ppm; MS/MS tolerance 0.8 Da.

### 4.7. Validation of DARTS Results via Immunoblotting

The samples obtained from the previously described experiments were submitted to Western blotting analysis. First, 7 µg of each sample were loaded on an 8% SDS-PAGE and transferred onto a nitrocellulose membrane; then, they were incubated for 1 h in a blocking solution (5% milk) and left for 16 h at 4 °C with monoclonal antibodies against PYGB (1:1000, Thermofisher, Invitrogen). Then, a mouse peroxidase-conjugated secondary antibody (1:1000) was added, and the signal was detected using an enhanced chemiluminescent substrate and LAS 4000 digital imaging system. Finally, an antibody against GAPDH (1:2500) in 5% milk has been tested as a loading normalizer. For the uncropped images see Figure S6.

### 4.8. t-LiP-MRM Analysis

An in-silico search for investigating PYGB tryptic peptides using the bio-informatics tool Peptide Atlas has been optimized to write appropriate MRM methods, setting the best MRM transitions of the theoretical PYGB fully tryptic peptides to map the protein sequences. Then, samples treated or not with 1g and submitted or not to the double-digestion procedure were analyzed by LC-MRM-MS to quantify as many fully tryptic peptides as possible for these proteins. 

### 4.9. Molecular Docking Analysis

The molecular docking procedure was performed, in order to identify the most probable binding complex between the human PYGB (pdbID: 5ikp) [45] and the 1g molecule, following the method previously reported by [46] and using the online docking web server SwissDock [47] as the docking algorithm. All parameters were set as default. The final complex geometry was rendered by PyMol software (The PyMOL Molecular Graphics System, Version 2.0, Schrödinger, LLC., Cambridge, MA, USA), whereas the 2D representation was created using the PoseView server [48]. 

### 4.10. Activity Assay 

U87MG was suspended in the lysis buffer (PBS pH 6.9, 0.1% Igepal, 1x Protease Inhibitors Cocktail) and then lysed carrying out three cycles of homogenization (1′on/1′off; on ice). The obtained proteome (10,000 rpm; 4 °C; 5′ in Eppendorf Centrifuge 5424-R) was quantified by Bradford spectrophotometric assay and diluted with PBS pH 6.9 containing glycerol to reach 10% of the disaggregating agent in the protein mixtures. A reaction mix, containing all the enzymes and the substrates (50% glycogen, 16 mM NADP, 500 µM glucose-1,6-diphosphate, 0.02 U/µL glucose-6-phosphate dehydrogenase, 0.02 U/µL phosphoglucomutase) involved in the combined reactions to follow the PYGB-dependent glycogenolysis, was prepared in PBS pH 6.9, 200 mM EDTA, 1M Magnesium acetate Mg (OAc) [46]. U87MG cells lysate and the reaction mix have been blended with and without AMP. The mixture has been divided into three aliquots: two of them have been treated with 1g (50 µM and 250 µM), while the last one served as control without 1g. All the samples have been incubated for 15′ (37 °C; 300 rpm) and then transferred onto a 384-multiwell plate (in duplicate; 30 µL for each replicate). The absorbance of the produced NADPH has been monitored in a kinetic mode for 200’ using the MultiskanGO spectrophotometer by Thermo Scientific (37 °C, orbital shaking: medium intensity, 10″ on/10″ off; λ = 340 nm; 1 scan/5′).

### 4.11. Statistical Analysis

All data are presented as the mean ± SD of at least three different experiments done in triplicate or quadruplicate. Differences between the treated cells versus control cells were analyzed using one-way analysis of variance ANOVA with Dunnett’s post-test, as indicated in the figures (Graph-Pad 9 Software Inc.). *p* values < 0.05 were considered significant.

## 5. Conclusions

This study has been mostly focused on the investigation of the interaction and action mechanism of 1g towards PYGB and its related pathways: a deeper analysis is necessary to better characterize the pharmacological profile of 1g, mainly in terms of pharmacokinetic and pharmacodynamic profile. In conclusions, our research highlights PYGB as a potential therapeutic target in GBM proposing 1g as a lead compound for developing a new class of simplified analogs active as anticancer drugs for glioblastoma, keeping unchanged its ability to cross the blood–brain barrier.

## Data Availability

The mass spectrometry proteomics data have been deposited to the ProteomeXchange Consortium via the PRIDE partner repository with the dataset identifier PXD034933.

## Supplementary Materials

The supporting information can be downloaded at: https://www.mdpi.com/article/10.3390/ijms23158200/s1.
