# Supplementary material for: A Proteomic Platform Unveils the Brain Glycogen Phosphorylase as a Potential Therapeutic Target for Glioblastoma Multiforme
Source: Int J Mol Sci. 2022 Jul 25;23(15):8200. doi: 10.3390/ijms23158200 (PMC9331883; doi:10.3390/ijms23158200)
Supplement: Supplementary file 1 [file ijms-23-08200-s001.zip › ijms-1812685-supplementary.pdf]

International Journal of *Molecular Sciences*

MDPI

Article

A proteomic platform unveils the brain glycogen phosphorylase as a potential therapeutic target for glioblastoma multiforme

Giusy Ferraro[1,2], Matteo Mozzicafreddo[3], Roberta Ettari[4], Lorenzo Corsi[5,6]* and Maria Chiara Monti[1]*

[1]Department of Pharmacy, University of Salerno, 84084 Fisciano, Italy
[2]PhD Program in Drug Discovery and Development, Department of Pharmacy, University of Salerno, 84084 Fisciano, Italy
[3]Department of Clinical and Molecular Sciences, Università Politecnica delle Marche, 60126 Ancona, Italy
[4]Department of Chemical, Biological, Pharmaceutical and Environmental Sciences, University of Messina, 98168, Messina, Italy
[5]Department of Life Sciences, University of Modena and Reggio Emilia, 41125 Modena, Italy
[6]INBB (Istituto Nazionale di Biostrutture e Biosistemi), 00136 Roma, Italy
* Correspondence: MCM mcmonti@unisa.it; LC lorenzo.corsi@unimore.it

		DARTS 1	Scores			
**Accession**	**Mass**	**Description**	**CTRL**	**1g 1uM**	**1g 10uM**	**1g 100uM**
Q9P273	305'093	Teneurin-3	0	0	0	103
Q14315	293'407	Filamin-C	59	78	63	87
Q9Y490	271'766	Talin-1	61	64	73	61
Q9NZM1	236'100	Myoferlin	0	0	0	49
Q15075	163'337	Early endosome antigen 1	87	100	115	115
O75116	161'939	Rho-associated protein kinase 2	0	46	0	57
Q7Z478	156'222	ATP-dependent RNA helicase DHX29	0	0	0	71
Q96J66	155'855	ATP-binding cassette sub-family C member 11	0	0	0	80
Q92878	154'823	DNA repair protein RAD50	65	67	86	95
O75976	153'919	Carboxypeptidase D	0	81	71	110
Q7L576	146'742	Cytoplasmic FMR1-interacting protein 1	92	100	104	112
O15067	146'297	Phosphoribosylformylglycinamidine synthase	43	57	43	61
P41252	145'718	Isoleucine--tRNA ligase, cytoplasmic	89	91	107	100
O15118	144'868	NPC intracellular cholesterol transporter 1	0	82	100	0
Q00341	141'995	Vigilin	0	0	0	64
P26640	141'642	Valine--tRNA ligase	60	78	93	60
Q5JPE7	140'435	Nodal modulator 2	0	0	100	0
P29144	139'745	Tripeptidyl-peptidase 2	52	70	64	82
Q92896	138'341	Golgi apparatus protein 1	33	80	60	83
O95373	120'751	Importin-7	40	57	73	27
Q9Y4F9	119'414	Rho family-interacting cell polarization regulator 2	0	142	150	0
P05023	114'135	Sodium/potassium-transporting ATPase subunit alpha-1	57	57	68	57
Q15029	110'336	116 kDa U5 small nuclear ribonucleoprotein component	0	0	62	54
P06400	106'947	Retinoblastoma-associated protein	0	0	136	0
Q7KZF4	102'618	Staphylococcal nuclease domain-containing protein 1	0	47	53	0
P11586	102'180	C-1-tetrahydrofolate synthase, cytoplasmic	0	0	31	0
Q13200	100'877	26S proteasome non-ATPase regulatory subunit 2	30	43	44	28
P11216	97'319	Glycogen phosphorylase, brain form	0	0	0	16
Q93050	97'148	V-type proton ATPase 116 kDa subunit a1	0	0	67	0
P55072	89'950	Transitional endoplasmic reticulum ATPase	0	0	12	10
O60645	85'969	Exocyst complex component 3	0	0	0	67
P16070	82'001	CD44 antigen OS=Homo sapiens	0	0	22	33
P54652	70'263	Heat shock-related 70 kDa protein 2	61	74	107	78
O60506	69'788	Heterogeneous nuclear ribonucleoprotein Q	0	0	11	14
P35241	68'635	Radixin	0	0	52	0
Q8N1G4	64'004	Leucine-rich repeat-containing protein 47	0	0	100	0
P30101	57'146	Protein disulfide-isomerase A3	0	0	50	0
P68363	50'804	Tubulin alpha-1B chain	0	0	100	0

		DARTS 2	Scores			
**Accession**	**Mass**	**Description**	**CTRL**	**1g 1uM**	**1g 10uM**	**1g 100uM**
Q8WXI7	1'520'295	Mucin-16	97	131	134	128
Q5T4S7	580'547	E3 ubiquitin-protein ligase UBR4	0	0	93	0
Q14204	534'809	Cytoplasmic dynein 1 heavy chain 1	81	85	103	129
Q07954	523'150	Prolow-density lipoprotein receptor-related protein 1	98	105	110	128
P78527	473'749	DNA-dependent protein kinase catalytic subunit	0	0	63	74
Q9P273	305'093	Teneurin-3	0	0	112	0
Q01082	275'237	Spectrin beta chain, non-erythrocytic 1	0	0	0	71
P24821	246'345	Tenascin	0	0	85	76
P35579	227'646	Mysion 9	48	65	83	88
P42694	220'601	Probable helicase with zinc finger domain	0	0	0	114
Q8TF72	218'321	Protein Shroom3 OS=Homo sapiens	0	0	75	0
O14647	212'183	Chromodomain-helicase-DNA-binding protein 2	0	118	147	0
Q6P0Q8	197'569	Microtubule-associated serine/threonine-protein kinase	0	0	103	97
Q9H792	195'039	Inactive tyrosine-protein kinase PEAK1	0	0	0	93
Q86UP2	156'464	Kinectin	0	0	0	33
Q56UN5	151'982	Mitogen-activated protein kinase kinase kinase 19	0	0	90	65
O15067	146'297	Phosphoribosylformylglycinamidine synthase	0	0	0	150
Q92896	138'341	Golgi apparatus protein 1 OS=Homo sapiens	52	52	59	59
O95347	136'085	Structural maintenance of chromosomes protein 2	0	0	143	162
Q12768	135'113	WASH complex subunit 5	0	0	62	0
Q7Z5K2	134'516	Wings apart-like protein homolog	0	0	0	136
O43847	132'644	Nardilysin	0	0	55	0
Q16531	128'142	DNA damage-binding protein 1	85	90	95	90
Q9NQ38	124'343	Serine protease inhibitor Kazal-type 5	0	0	0	76
P53396	121'674	ATP-citrate synthase	0	0	0	47
**O95373**	120'751	Importin-7	0	0	36	33
P06756	117'048	Integrin alpha-V	58	58	69	73
Q6IEE7	116'819	Transmembrane protein 132E	0	0	57	0
Q7Z410	116'115	Transmembrane protease serine 9	0	0	164	0
Q02413	114'702	Desmoglein-1	0	50	114	121
P12109	109'602	Collagen alpha-1(VI) chain	0	0	53	71
P36776	106'936	Lon protease homolog, mitochondrial	0	0	106	0
P12814	103'563	Alpha-actinin-1	0	0	0	82
P27987	103'167	Inositol-trisphosphate 3-kinase B	0	0	0	79
Q14974	98'420	Importin subunit beta-1	0	0	0	109
P11216	97'319	Glycogen phosphorylase, brain form	0	0	0	78
P34932	95'127	Heat shock 70 kDa protein 4	0	0	38	0
P55072	89950	Transitional endoplasmic reticulum ATPase	200	286	248	290
Q15436	87018	Protein transport protein Sec23A	0	0	0	120
Q01813	86454	ATP-dependent 6-phosphofructokinase, platelet type	119	108	142	150
Q99798	86113	Aconitate hydratase, mitochondrial	156	256	156	222
O00469	85'373	Procollagen-lysine,2-oxoglutarate 5-dioxygenase 2	0	0	0	58
P07900	85'006	Heat shock protein HSP 90-alpha	0	0	100	100
Q96G03	68'754	Phosphoglucomutase-2	52	90	95	105
P38606	68'660	V-type proton ATPase catalytic subunit A	94	135	147	147
P27824	67'982	Calnexin	0	0	0	47
Q9NSD9	66'701	Phenylalanine--tRNA ligase beta subunit	0	0	0	55
Q8TF66	65'238	Leucine-rich repeat-containing protein 15	0	58	67	50
O15371	64'560	Eukaryotic translation initiation factor 3 subunit D	0	0	0	85
P31948	63'227	Stress-induced-phosphoprotein 1	44	42	45	61
O14773	61'723	Tripeptidyl-peptidase 1	0	0	75	100
P04062	60'134	Lysosomal acid glucosylceramidase	0	60	100	100
Q5VTE0	50'495	Putative elongation factor 1-alpha-like 3	0	108	92	115

		DARTS 3	Scores			
**Accession**	**Mass**	**Description**	**CTRL**	**1g 1uM**	**1g 10uM**	**1g 100uM**
Q07954	523'150	Prolow-density lipoprotein receptor-related protein 1	61	113	66	74
P78527	473'749	DNA-dependent protein kinase catalytic subunit	0	0	43	0
Q01082	275'237	Spectrin beta chain, non-erythrocytic 1	100	123	110	85
P35579	227'646	Myosin-9 OS=Homo sapiens	47	59	60	51
Q02952	191'937	A-kinase anchor protein 12	0	0	0	110
Q08378	167'765	Golgin subfamily A member 3	0	16	32	0
Q14152	166'867	Eukaryotic translation initiation factor 3 subunit A	0	0	0	7
Q9BZF9	163'545	Uveal autoantigen with coiled-coil domains and ankyrin r	0	0	47	0
Q08211	142'181	ATP-dependent RNA helicase A	0	61	55	50
Q00341	141'995	Vigilin	63	91	69	80
P26640	141'642	Valine--tRNA ligase	76	78	82	53
Q15393	136'575	Splicing factor 3B subunit 3	45	55	68	36
Q9P2J5	135'577	Leucine--tRNA ligase, cytoplasmic OS=Homo sapiens OX=!	0	0	29	0
Q16531	128'142	DNA damage-binding protein 1	61	82	71	73
Q12965	127'552	Unconventional myosin-Ie	0	41	0	0
Q92900	125'578	Regulator of nonsense transcripts 1	45	106	97	100
O60566	120'781	Mitotic checkpoint serine/threonine-protein kinase BUB1	0	0	100	0
O95373	120'751	Importin-7	0	73	91	0
P53992	119'789	Protein transport protein Sec24C	0	0	29	0
Q8N7X1	115'724	RNA-binding motif protein, X-linked-like-3	52	100	122	0
O94855	114'476	Protein transport protein Sec24D	0	0	30	0
O43592	111'148	Exportin-T	0	0	0	38
O60763	108'740	General vesicular transport factor p115	0	0	0	20
Q99460	106'795	26S proteasome non-ATPase regulatory subunit 1	0	48	39	52
Q99613	105'962	Eukaryotic translation initiation factor 3 subunit C	0	0	0	73
P52789	103'739	Hexokinase-2	60	62	63	54
P12814	103'563	Alpha-actinin-1	560	580	400	620
P35606	103'278	Coatomer subunit beta	36	46	54	36
Q7KZF4	102'618	Staphylococcal nuclease domain-containing protein 1	633	633	700	683
P11586	102'180	C-1-tetrahydrofolate synthase, cytoplasmic	0	45	27	73
P11216	97'319	Glycogen phosphorylase, brain form	200	250	175	275
Q96QK1	92'447	Vacuolar protein sorting-associated protein 35	48	52	62	52
P55072	89'950	Transitional endoplasmic reticulum ATPase	350	406	344	478
P42224	87'850	Signal transducer and activator of transcription 1-alpha/be	0	22	67	67
Q01813	86'454	ATP-dependent 6-phosphofructokinase, platelet type	63	67	79	96
Q12797	86'266	Aspartyl/asparaginyl beta-hydroxylase	0	0	33	50
P49589	86'103	Cysteine--tRNA ligase, cytoplasmic	0	80	0	100
P08237	85'984	ATP-dependent 6-phosphofructokinase, muscle type	73	82	82	100
Q96TA1	84'598	Protein Niban 2	0	13	42	13
P41250	83'854	Glycine--tRNA ligase	90	94	94	106
P22033	83'538	Methylmalonyl-CoA mutase, mitochondrial	0	0	67	33
Q12996	83'325	Cleavage stimulation factor subunit 3	0	0	0	200
Q9NY33	82'880	Dipeptidyl peptidase 3	106	135	118	129
P13798	82'142	Acylamino-acid-releasing enzyme	80	100	120	127
P48147	81'560	Prolyl endopeptidase	29	43	71	86
O95573	81'338	Long-chain-fatty-acid--CoA ligase 3	36	36	64	64
P43304	81'315	Glycerol-3-phosphate dehydrogenase, mitochondrial	0	0	100	0
Q8NBF2	80'249	NHL repeat-containing protein 2	0	0	13	13
O60488	80'220	Long-chain-fatty-acid--CoA ligase 4	121	129	114	179
Q06210	79'555	Glutamine--fructose-6-phosphate aminotransferase [ison	0	0	36	0
P33121	78'919	Long-chain-fatty-acid--CoA ligase 1	0	0	143	157
P08253	74'918	72 kDa type IV collagenase	0	50	67	100
P38646	73'920	Stress-70 protein, mitochondrial	90	92	122	115
Q9H4A4	73'234	Aminopeptidase B	63	75	81	88
Q13409	71'811	Cytoplasmic dynein 1 intermediate chain 2	44	67	100	100
O43390	71'184	Heterogeneous nuclear ribonucleoprotein R	26	26	43	39
P49748	70'745	Very long-chain specific acyl-CoA dehydrogenase, mitoch	100	111	106	111
Q9NQW7	70'558	Xaa-Pro aminopeptidase 1	75	75	106	131
P54652	70'263	Heat shock-related 70 kDa protein 2	78	80	108	88
P17844	69'618	Probable ATP-dependent RNA helicase DDX5	58	63	54	75
P43378	68'547	Tyrosine-protein phosphatase non-receptor type 9	0	43	243	257
Q9BYC5	66'930	Alpha-(1,6)-fucosyltransferase	80	80	90	120
Q07866	65'782	Kinesin light chain 1	0	0	21	32
Q96AY3	64'717	Peptidyl-prolyl cis-trans isomerase FKBP10	140	140	160	100
P21589	63'898	5'-nucleotidase	100	107	80	113
Q13057	62'632	Bifunctional coenzyme A synthase	63	63	75	75
Q96L92	61'854	Sorting nexin-27	0	60	60	60
P10155	61'372	60 kDa SS-A/Ro ribonucleoprotein	79	89	89	63
Q9H857	61'022	5'-nucleotidase domain-containing protein 2	0	100	67	100
Q15392	60'803	Delta(24)-sterol reductase	0	50	0	75
Q16222	59'131	UDP-N-acetylhexosamine pyrophosphorylase	54	69	54	69
Q92692	58'162	Nectin-2	125	125	125	175
P49257	57'798	Protein ERGIC-53	30	52	30	70
P30837	57'626	Aldehyde dehydrogenase X, mitochondrial	57	64	71	86
Q9NNW7	57'156	Thioredoxin reductase 2, mitochondrial	100	125	125	75
O94788	57'144	Retinal dehydrogenase 2	0	78	89	0
Q15758	57'018	Neutral amino acid transporter B(0)	33	67	67	100
Q9UHG3	57'003	Prenylcysteine oxidase 1	85	92	85	108
Q9Y3I0	55'688	RNA-splicing ligase RtcB homolog	119	138	106	138
Q13509	50'856	Tubulin beta-3 chain	0	0	13	0
Q9Y265	50'538	RuvB-like 1	35	45	45	50

Figure S1: For each DARTS experiment, it has been reported the entire list of proteins which are protected by 1g at least at one concentration, together with their Mascot Score.

Accession	Score	Mass	Matches	Match(sig)	Sequences	Seq(sig)	emPAI	Description
P11216	281	97319	25	11	25	11	0.71	Glycogen phosphorylase, brain form
**Accession**	**Score**	**Mass**	**Matches**	**Match(sig)**	**Sequences**	**Seq(sig)**	**emPAI**	**Description**
P55072	354	89950	51	18	39	16	1.45	Transitional endoplasmic reticulum ATPase
**Accession**	**Score**	**Mass**	**Matches**	**Match(sig)**	**Sequences**	**Seq(sig)**	**emPAI**	**Description**
O95373	291	120751	33	14	25	13	0.67	Importin-7

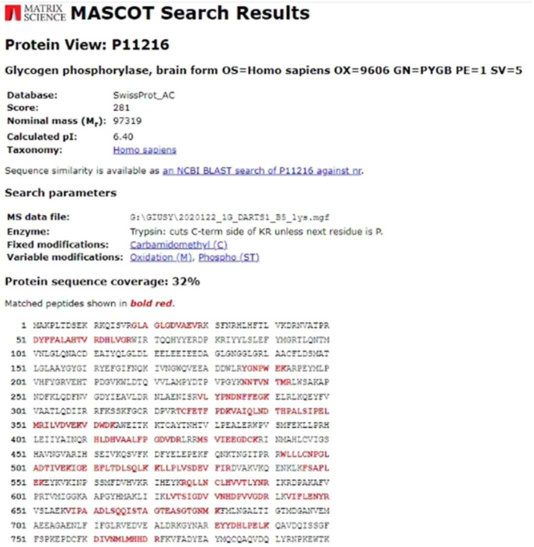

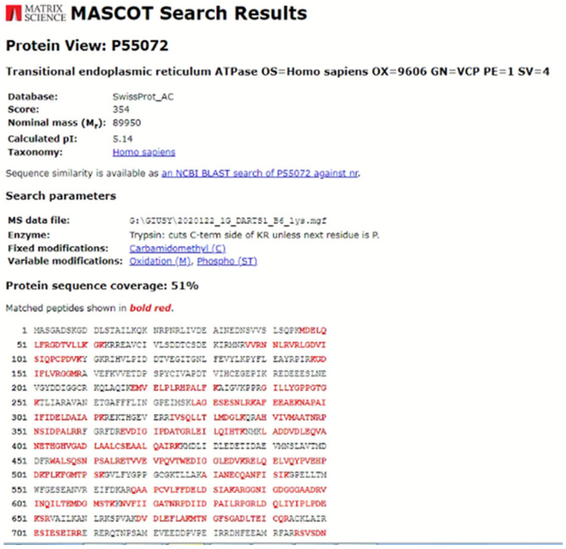

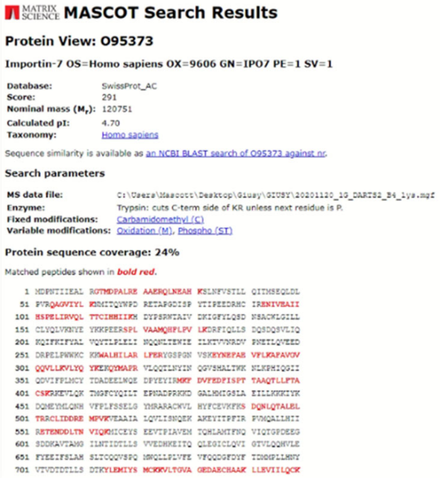

Figure S2: By way of example, it is reported the full Mascot output for the identification of the three protected proteins in the cell lysate, together with the protein sequence coverage percentage.

Q1_mz	Q3_mz	Sequence	ID	R.T. (min)
249.40	203.13	HLHFTLVK	H[35-42]K	3.28
447.23	457.76	DYFFALAHTVR	D[51-61]R	4.67
375.51	467.23	TQQHYYER	T[71-78]R	3.75
777.89	802.36	IYYLSLEFYMGR	I[83-94]R	7.21
573.29	275.17	YEFGIFNQK	Y[162-170]K	3.71
915.45	775.37	IVNGWQVEEADDWLR	I[171-185]R	7.43
447.21	559.29	YGNPWEK	Y[186-192]K	4.64
434.72	542.27	ARPEYMLPVHFYGR	A[193-206]R	3.77
475.23	721.37	NNTVNTMR	N[236-243]R	5.82
933.97	290.15	LQDFNVGDYIEAVLDR	L[255-270]R	7.38
721.85	615.78	VLYPNDNFFEGK	V[279-290]K	5.36
572.75	359.19	TC[160]FETFPDK	T[325-333]K	5.64
706.72	563.82	VAIQLNDTHPALSIPELMR	V[334-352]R	5.4
408.25	589.32	ILVDVEK	I[353-359]K	5.23
625.64	357.69	TC[160]AYTNHTVLPEALER	T[372-387]R	4.95
512.25	837.42	WPVSMFEK	W[388-395]K	4.68
457.26	560.31	HLEIIYAINQR	H[400-410]R	5.36
554.62	658.32	HLDHVAALFPGDVDR	H[411-425]R	5.95
505.52	516.29	INMAHLC[160]VIGSHAVNGVAR	I[440-458]R	5.78
275.83	288.17	IHSEIVK	I[459-465]K	3.33
585.27	373.21	DFYELEPEK	D[471-479]K	5.6
921.50	375.22	WLLLC[160]NPGLADTIVEK	W[492-507]K	7.47
700.91	964.51	LLPLVSDEVFIR	L[522-533]R	7.23
421.23	389.24	FSAFLEK	F[546-552]K	3.62
581.65	242.15	QLLNC[160]LHVVTLYNR	Q[577-590]R	4.83
353.20	374.24	TVMIGGK	T[603-609]K	3.36
315.83	218.15	AAPGYHMAK	A[610-618]K	3.45
631.33	840.42	LVTSIGDVVNHDPVVGDR	L[623-640]R	5.62
527.29	581.27	VIFLENYR	V[643-650]R	3.2
436.22	589.3	EYYDHLPELK	E[731-740]K	4.61
755.88	769.39	QAVDQISSGFFSPK	Q[741-754]K	7.07
345.92	290.15	DIVNMLMHHDR	D[761-771]R	3.75
375.18	522.23	NIAC[160]SGK	N[805-811]K	4.09
427.22	639.31	TITEYAR	T[817-823]R	4.22
719.72	347.21	EIWGVEPSDLQIPPPNIPR	E[824-842]R	5.95

Figure S3: Here the list of the tryptic peptides of PYGB found by LiP-MS has been reported together with the m/z value, the m/z value of the best fragment, the primary sequence, the ID on the protein sequence and the r.t. in the UPLC trace.

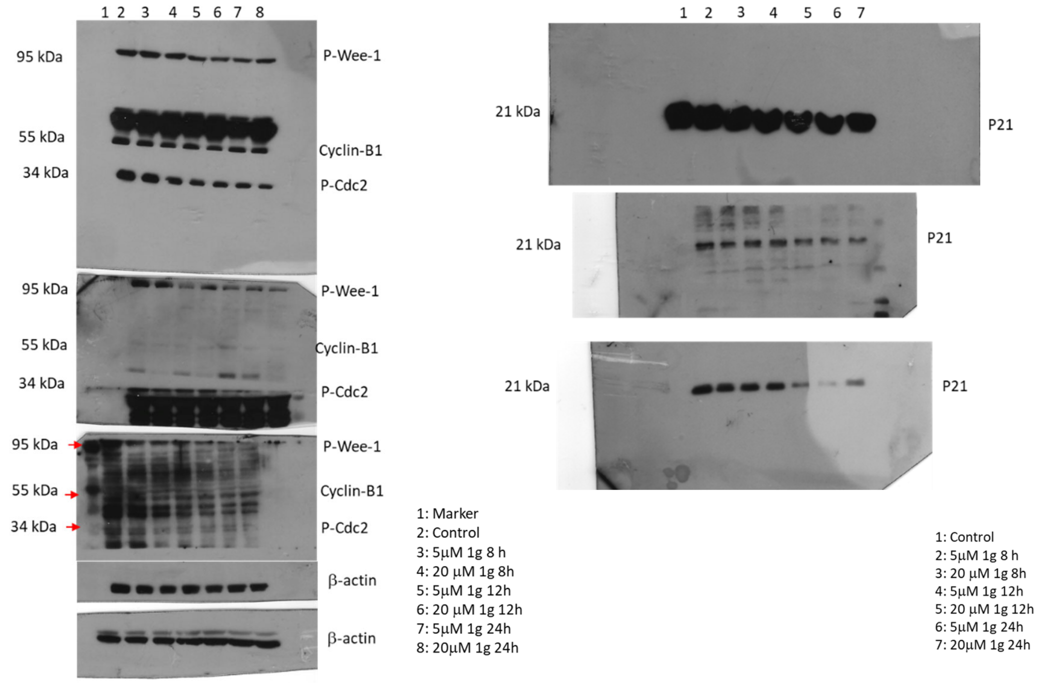

Figure S4: Uncropped Western Blotting experiment as reported in Figure 2A

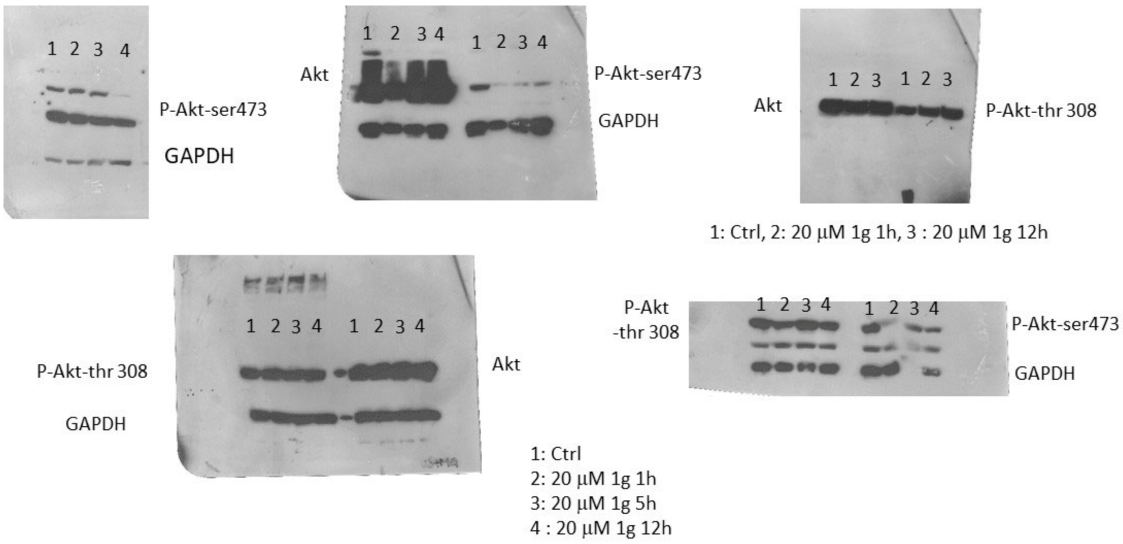

Figure S5: Uncropped Western Blotting experiment as reported in Figure 2C

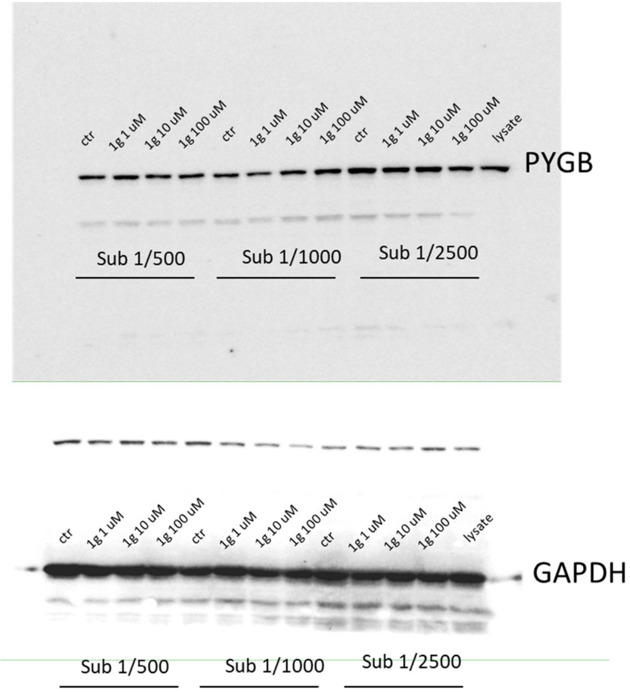

Figure S6: Uncropped Western Blotting experiment as reported in Figure 3E

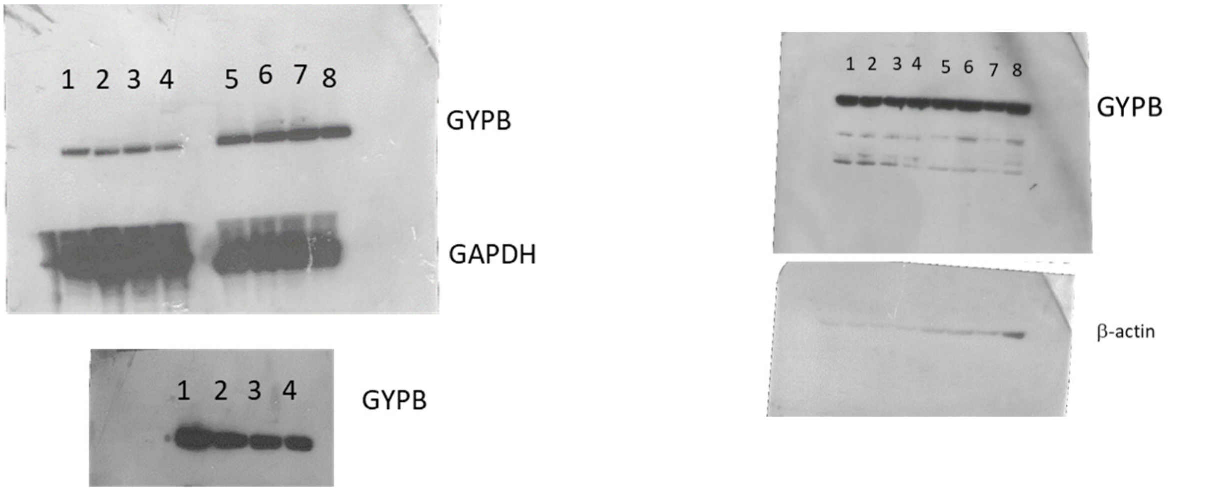

1: Ctrl, 2: 20 μM 1g 5h, 3: 20 μM 1g 12h
4 : 20 μM 1g 24h, 5: 20 μM 1g 24h, 6: 20 μM 1g 12h
7: 20 μM 1g 5h, 8: Ctrl

1: Ctrl, 2: 20 μM 1g 5h, 3: 20 μM 1g 12h
4 : 20 μM 1g 24h, 5: Ctrl, 6: 20 μM 1g 12h
7: 20 μM 1g 24h, 8: Ctrl

Figure S7: Uncropped Western Blotting experiment as reported in Figure 5B
